# *Ageratina adenophora* Disrupts the Intestinal Structure and Immune Barrier Integrity in Rats

**DOI:** 10.3390/toxins13090651

**Published:** 2021-09-15

**Authors:** Yujing Cui, Samuel Kumi Okyere, Pei Gao, Juan Wen, Suizhong Cao, Ya Wang, Junliang Deng, Yanchun Hu

**Affiliations:** Key Laboratory of Animal Diseases and Environmental Hazards of Sichuan Province, College of Veterinary Medicine, Sichuan Agricultural University, Chengdu 611130, China; yjchoi@163.com (Y.C.); samuel20okyere@gmail.com (S.K.O.); gaopejune@163.com (P.G.); juanwen881010@163.com (J.W.); suizhongcao@126.com (S.C.); wangyayang@126.com (Y.W.)

**Keywords:** *Ageratina adenophora*, pathological changes, intestinal immune barrier, inflammation cytokines

## Abstract

The aim of this study was to investigate the effects of *Ageratina adenophora* on the intestines morphology and integrity in rat. Rats were randomly divided into two groups and were fed with 10 g/100 g body weight (BW) basal diet and 10 g/100 g BW experimental diet, which was a mixture of *A. adenophora* powder and basal diet in a 3:7 ratio. The feeding experiment lasted for 60 days. At days 28 and 60 of the experiment, eight rats/group/timepoint were randomly selected, weighed, and sacrificed, then blood and intestinal tissues were collected and stored for further analysis. The results showed that *Ageratina adenophora* caused pathological changes and injury in the intestine, elevated serum diamine oxidase (DAO), D-lactate (D-LA), and secretory immunoglobulin A (sIgA) levels, reduced occludin levels in intestinal tissues, as well as increased the count of intraepithelial leukocytes (IELs) and lamina propria leukocytes (LPLs) in the intestine (*p* < 0.05 or *p* < 0.01). In addition, the mRNA and protein (ELISA) expressions of pro-inflammation cytokines (IL-1β, IL-2, TNF-α, and IFN-ϒ) were elevated in the *Ageratina adenophora* treatment groups, whereas anti-inflammatory cytokines such as IL-4 and IL-10 were reduced (*p* < 0.01 or *p* < 0.05). Therefore, the results obtained in this study indicated that *Ageratina adenophora* impaired intestinal function in rats by damaging the intestine structure and integrity, and also triggered an inflammation immune response that led to intestinal immune barrier dysfunction.

## 1. Introduction

*Ageratina adenophora* (Spreng.) R.M. King and H. Rob., commonly known as Crofton weed, is an invasive plant which is native to Mexico and Central America; however, it is broadly found in other parts of the world including China [1,2]. *A. adenophora* has been identified to be toxic to most farm animals and humans. Recent studies have reported various toxic effects of *A. adenophora* on various organs in different animal models (such as cattle, horse, rodent, sheep, and goats) that ingested this plant [3,4,5]. Sun et al. [6] reported that *A. adenophora* could cause liver toxicity in mice, whereas other reports indicated that *A. adenophora* could induced spleen toxicity in mice [7]. In goats, *A. adenophora* induced oxidative stress in renal cells and also caused G1 arrest in the kidney cells [8]. Moreover, other studies indicated that *A. adenophora* causes numerous effects in animals including livestock poisonings, leading acute asthma, diarrhea, depilation, bile duct hyperplasia, and even death [9]. Organ toxicity by numerous secondary metabolites derived from *A. adenophora,* such as 9-oxo-10,11-dehydro-agerophorone (ODA), 2-deoxo-2-(acetyloxy)-9-oxo- ageraphorone (DAOA), and 9-oxo-agerophorone (OA), have been reported [10,11,12]. In mice and rat models, ODA was reported as the main toxic agent that induced liver lesions [12,13,14]. A current study by Okyere et al. [15] reported that ODA caused apoptosis and G0/G1 cell arrest of hepatocytes in mice. In addition, in mouse splenocytes, ODA induced G1 arrest and autophagy [16].

The gastrointestinal tract plays a major role in food digestion, assimilation, and health in both humans and animals [17]. The intestinal epithelial layer forms the major barrier that protects our body from ingested toxins, bacteria, and their metabolites [18]. An imbalance in the intestinal barrier structure could lead to an uncontrolled immune reaction in the intestinal microenvironment, which may further cause various diseases, including intestinal inflammatory disorders, extra-intestinal autoimmune diseases, such as rheumatoid arthritis and multiple sclerosis, and metabolic disorders such as diabetes and obesity [19,20]. Therefore, intestinal barrier permeability may be a prognostic marker for disease pathophysiology. *A. adenophora* has been reported to cause congestion in the intestine and gastric mucosa [21]. However, these results did not provide an in-depth information about the toxicity of *A. adenophora* on intestinal health, hence, this topic requires further studies.

Therefore, this study aimed to investigate the toxic effects of *A. adenophora* ingestion on intestinal health, specifically on the intestinal structure and immune barrier integrity of rats.

## 2. Results

### 2.1. A. adenophora Administration Increased Intestinal Pathological Injury in Rat

#### 2.1.1. Effects of *A. adenophora* on Histopathological Changes in the Small Intestinal Sections of Rats

**Figure 1:**
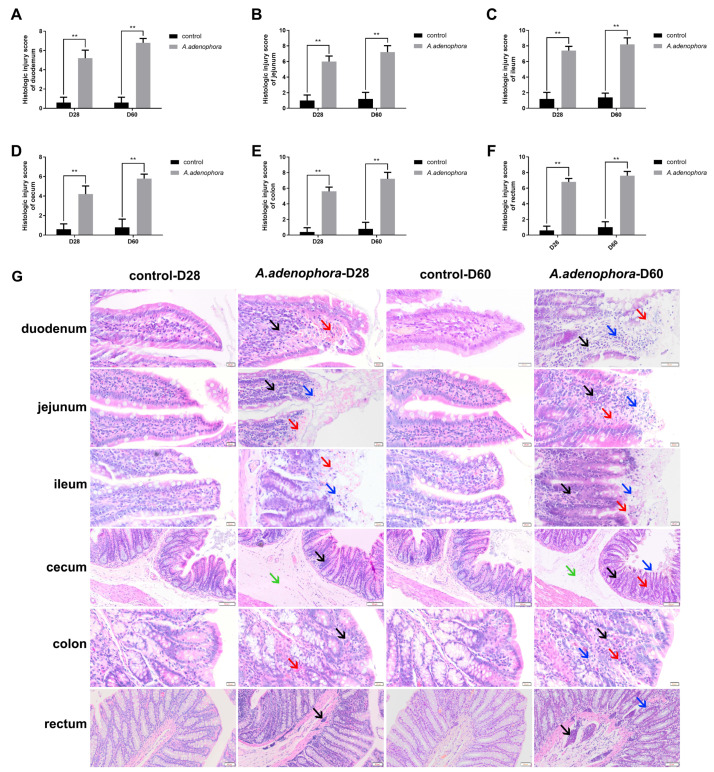
Histopathological observation of in the intestinal sections of rats from the control and *A. adenophora* groups after 60 days experimental feeding. (**A**–**F**) Histologic injury score of intestinal sections. Bars represent means ± SD for eight rats per treatment. Within the same day (D), bars with ** differ significantly (*p* < 0.01). Control = rats were fed only normal diet. *A. adenophora* = rats were fed a mixture of basal diet and leaf powder of *A. adenophora* at 7:3 ratio (**G**) Photograph of pathological changes in the various sections in the intestine. The *A. adenophora* treatment groups showed observable pathological changes characterized by lymphocytic proliferation, edema, necrosis, and inflammation; however, these were absent in the control groups. Inflammatory cells infiltration is indicated by black arrows, bleeds are indicated by red arrows, structural damage are indicated by blue arrows, and edema are indicated by green arrows.

From the histopathological staining we observed various pathological changes in the intestinal sections (Figure 1). The histological injury results showed a significant increase in tissue injury for all intestinal sections in the treatment group as compared to the control group (Figure 1A–F, *p* < 0.01).

As shown in Figure 1G, the small intestinal structure of the control group was complete at all the time points in this study. We observed that the thickness of serosa layer, muscular layer, and sub-mucosa were uniform. In addition, the lamina propria and epithelial layer were also clear, and the villi structures were complete and orderly arranged.

Compared with the control group, the duodenum of treatment groups showed mild edema, inflammatory cell infiltration, and hyperemia in lamina propria at D28. In addition, we also observed an increased villi width in the treatment groups compared to the control group at D28. However, at D60, as the feeding time increased and the degree of damage of the duodenum structure gradually increased, showing characteristics such as hemorrhagic edema in the mucosa and sub-mucosa and a large number of inflammatory cells infiltrating into the epithelial layer and lamina propria. We also observed damage of the villi, which included lodging, disordered arrangement, severe abscission of the local top, severe coagulation necrosis of epithelial layer, and loss of structure of the villi in the treatment group compared to the control at D60. The pathological results obtained in this study showed that villi structure erosion was the main pathological damage caused by *A. adenophora*.

The jejunum in the treatment group showed bleeding at the top of the villi. In addition, all the villi tips showed coagulation necrosis, formed translucent coagulation necrosis structures in the intestinal epithelium, and infiltrated lymphocytes in the intrinsic layer and epithelial layer at D28. Furthermore, compared with the control group at D28, we observed bleeding at the top of the ileum villi; also, the epithelial cells were exfoliated and necrotic in the treatment group. The exfoliated epithelial cells and red blood cells constituted a “pink ring” in the ileum. However, at D60, the degree of necrosis in the treatment groups jejunum increased. Furthermore, we observed that the columnar cells at the top of the villi diffused necrosis heavily, and the local villi tip was seriously exfoliated and shed off. In addition, we observed shorter intestinal villi, damaged crypt, and loss of villi structure in the treatment group. To add up, inflammatory cells infiltration occurred on a large area of sub-mucosa with bleeding.

Moreover, at D60, diffuse infiltration of dermatitis cells in the mucosa associated with bleeding, severe shedding of villi top, coagulative necrosis, complete disappearance of nuclei of exfoliated and necrotic epithelial cells, and formation of a translucent “inner ring” necrotic layer were observed in the ileums of the treatment groups. Therefore, the pathological results showed that *A. adenophora* could induce coagulative necrosis of jejunum and ileum in rats.

#### 2.1.2. Effects of *A. adenophora* on Histopathology of Large Intestinal Sections in Rats

As shown in Figure 1G, the structure of large intestinal sections of the control group were complete at all timepoints, and the sub-mucosa, muscle layer, and intestinal gland were arranged normally, and the morphology of lamina propria and epithelial layer were complete. In addition, the mucosa and muscle layer in the control group were clear at all timepoints.

Compared with the control group, the caecum of the treatment group showed sub-mucosal edema and mild local inflammatory infiltration of the epithelial layer at D28. However, at D60, the treatment groups showed thickening of the sub-mucous and muscular layer in edema. In addition, a large range of villous epithelial cells at the top of mucosal layer fell off with local necrosis, which exposed the lamina propria and damaged the crypt, resulting in sub-mucosa hyperemia and hemorrhage, infiltration of a small amount of inflammatory cells, and structural damage. These results signified that *A. adenophora* could cause injuries in the caecum of rats characterized by sub-mucosa edema.

Furthermore, compared to the treatment groups, we observed swelling of the colonic muscle layer, congestion, mild hemorrhage of lamina propria, and a large number of inflammatory cells infiltration in the epithelial layer at D28. Again, in addition to the observation at D28, the treatment groups showed capillary infiltration in the lamina propria and local loss of lamina propria at D60. Therefore, inflammatory infiltration was the main pathological feature of *A. adenophora* in the rats’ colon, and the degree of infiltration of inflammatory cell was linearly correlated with the feeding time.

In the rectum of the treatment groups, we observed a large number of lymphocytes infiltrated into the mucosa and sub-mucosa, as well as local lymphocyte proliferation at D28. However, at D60, we observed that the enteraden epithelial cells were swollen, and the epithelial layer and sub-mucosa were infiltrated with proliferative inflammatory cells. We also observed that the intestinal glands became shorter and the local lamina propria structure changed in the treatment groups. This pathological results showed that *A. adenophora* could induce a large number of rectal lymphocytes and lymphocyte proliferation in rats and that the degree of change was time dependent.

#### 2.1.3. Effect of *A. adenophora* on Villi Height, Crypt Depth, and the Villi Height Ratio/Crypt Depth (*v*/*c*) of the Small Intestine Sections in Rats

The villi height and the villi height ratio/crypt depth (*v*/*c*) of the duodenum, jejunum, and ileum sections of the control groups were significantly higher as compared to the treatment groups at each timepoint (D28 and D60, respectively) (*p* < 0.01) (Table 1). However, the crypt depth of the duodenum, jejunum, and ileum of the control groups was significantly lower as compared to the treatment groups at each timepoint, respectively (*p* < 0.01) (Table 1).

#### 2.1.4. Effects of *A. adenophora* on the IELS and LPLS Count of Intestinal Sections in Rats

**Figure 2:**
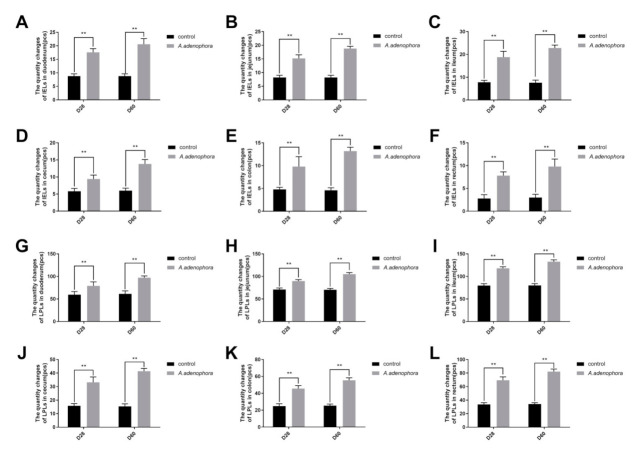
The intestinal intraepithelial lymphocytes (IELs) and lamina propria leukocytes (LPLs) counts in rats from the control and *A. adenophora* groups after 60 days of experimental feeding. (**A**–**F**) Intestinal intraepithelial lymphocytes (IELs) in intestines of rats. (**G**–**L**) Lamina propria leukocytes (LPLs) counts in various intestinal sections of rats. Bars represent means ± SD for eight rats per treatment. Within the same day (D), bars with ** differ significantly (*p* < 0.01). Control = rats were fed only basal diet. *A. adenophora* = rats were fed a mixture of basal diet and leaf powder of *A. adenophora* in a 7:3 ratio.

IELS count of all intestinal sections of the treatment groups were significantly higher as compared to that of the control group at each timepoint, respectively (*p* < 0.05) (Figure 2A–F). Moreover, the results obtained from the LPLS count also showed a significant increase in LPLS levels in the treatment groups as compared to the control group at each timepoint (*p* < 0.05) (Figure 2G–L).

### 2.2. A. adenophora Disrupts Intestinal Barrier Integrity

**Figure 3:**
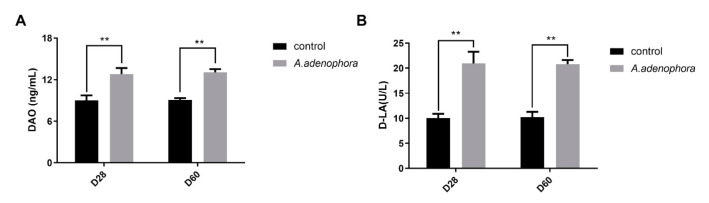
DAO and D-LA concentrations in rats’ serum from the control and *A. adenophora* groups after 60 days experimental feeding. (**A**) DAO levels in serum of rats. (**B**) D-LA levels in serum of mice. Bars represent means ± SD for 8 rats per treatment. Within the same day (D), bars with ** differ significantly (*p* < 0.01). Control = rats were fed only basal diet. *A. adenophora* = rats were fed a mixture of basal diet and leaf powder of *A. adenophora* at 7:3 ratio.

**Figure 4:**
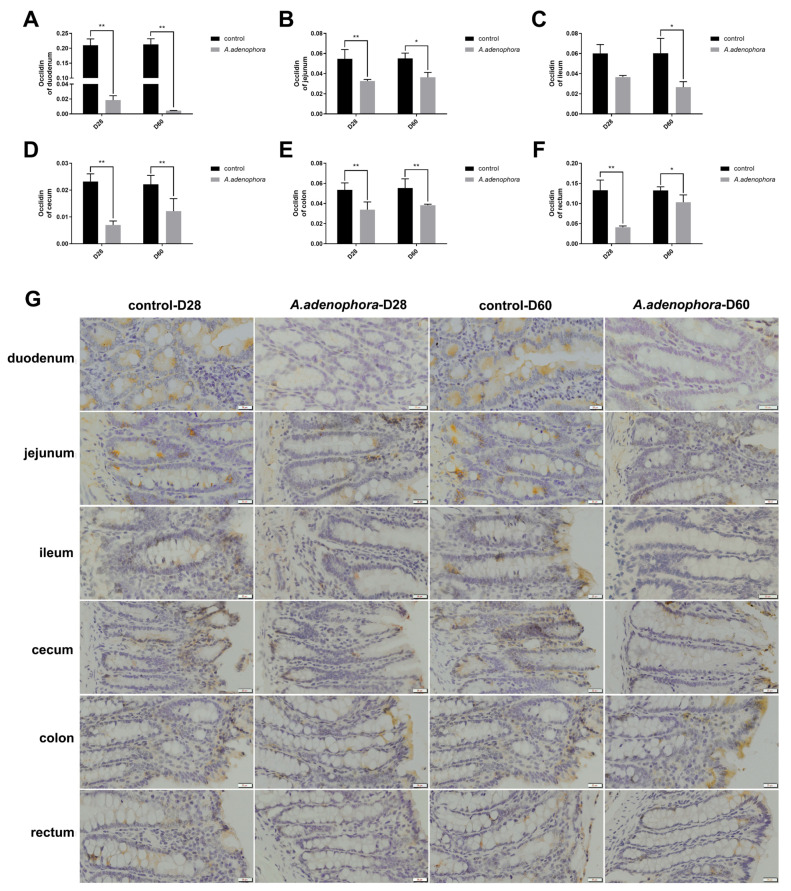
Occludin levels in the various section of rat intestine from the control and *A. adenophora* groups after 60 days of experimental feeding. (**A**–**F**) Occludin levels in various intestinal sections of rats. Bars represent means ± SD for eight rats per treatment. Within the same day (D), bars with ** or * differ significantly (*p* < 0.01 or *p* < 0.05). (**G**) Immunohistochemistry staining photographs of intestinal sections (yellow area represents occludin). Control = rats were fed only basal diet. *A. adenophora* = rats were fed a mixture of basal diet and leaf powder of *A. adenophora* in a 7:3 ratio.

To determine the effects of *A. adenophora* on intestinal integrity, we checked DAO and D-LA concentrations in the serum. In addition, occluding concentrations in various intestinal sections were determined. Our results showed that the DAO and D-LA levels in the serums of the *A. adenophora* treated rats were significantly higher compared to the controls at each timepoint, respectively (*p* < 0.05, Figure 3A,B). In addition, the occludin levels of the treatment groups were significantly lower as compared to those of the control groups at each timepoint, respectively (*p* < 0.05, Figure 4).

### 2.3. Effects of A. adenophora on the Concentration of Intestinal sIgA of Rats

**Figure 5:**
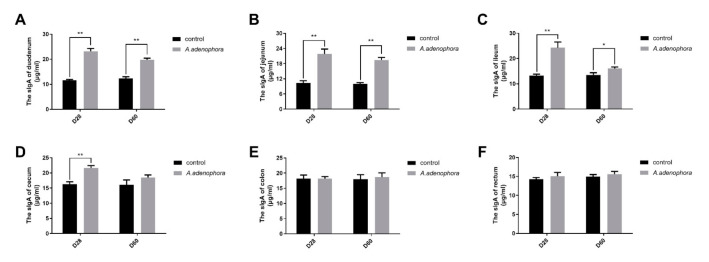
Intestinal mucosa sIgA levels in rats from the control and *A. adenophora* groups after 60 days of experimental feeding. (**A**–**F**) sIgA levels in various intestinal sections of rats. Bars represent means ± SD for eight rats per treatment. Within the same day (D), bars with ** or * differ significantly (*p* < 0.01 or *p* < 0.05). Control = rats were fed only basal diet. *A. adenophora* = rats were fed a mixture of basal diet and leaf powder of *A. adenophora* in a 7:3 ratio.

To investigate the immune action of *A. adenophora,* the sIgA levels in the intestinal mucosa of rats were determine using ELISA. The results showed that the concentration of sIgA in the small intestinal sections were higher in the treatment groups as compared to the control group at each timepoint (duodenum, jejunum, ileum D28; *p* < 0.01; ileum D60; *p* < 0.05, Figure 5A–C). However, in the large intestine, the sIgA concentration in the caecum sections of the treatment groups was higher compared to the control group at D28 (*p* < 0.05, Figure 5C). There were no significant differences in the colon or in the rectum sIgA levels at the D28 and D60 timepoints, even though the sIgA concentration values were higher in the treatment groups than in the control groups (*p* > 0.01 or *p* > 0.05, Figure 5E,F).

### 2.4. Expression Levels of Intestinal Tissue Pro- and Anti-Inflammation-Related Cytokines Levels

#### 2.4.1. mRNA and Protein (ELISA) Expressions of Pro and Anti-Inflammation-Related Cytokines in Duodenum of *A. adenophora*-Fed Rats

**Figure 6:**
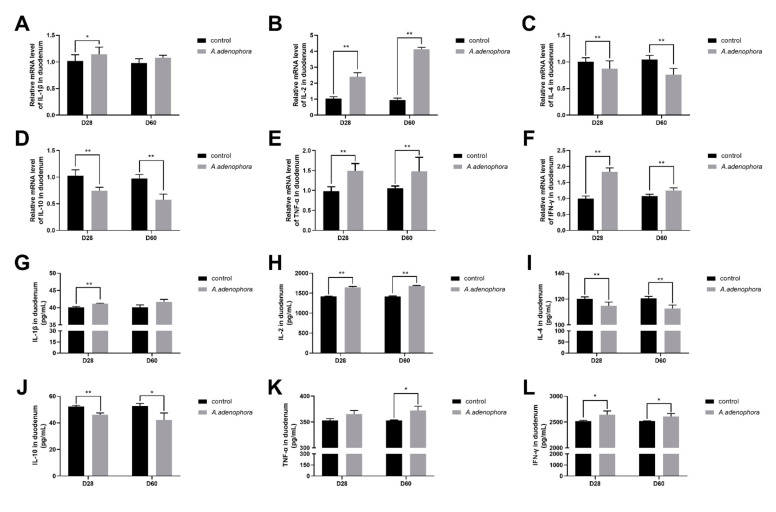
mRNA and protein (ELISA) expression levels of pro- and anti-inflammation-related cytokines in duodenum of rats from the control and *A. adenophora* groups after 60 days of experimental feeding. (**A**–**F**) mRNA expression of pro- and anti-inflammation cytokines. (**G**–**L**) Protein (ELISA) expression of pro- and anti-inflammatory cytokines. Bars represent means ± SD for eight rats per treatment. Within the same day (D), bars with ** or * differ significantly (*p* < 0.01 or *p* < 0.05). Control = rats were fed only basal diet. *A. adenophora* = rats were fed a mixture of basal diet and leaf powder of *A. adenophora* in a 7:3 ratio.

From the gene expression results, it was observed that the levels of pro-inflammatory cytokines (IL-1β, IL-2, TNF-α, and IFN-ϒ) were all increased in the *A. adenophora* treatment groups as compared to the control at all the timepoints, except that the gene expression levels of IL-1β showed no significant increase at D60 (*p* > 0.01 or *p* > 0.05). In addition, we also observed that the gene expression levels of anti-inflammatory cytokines (IL-4 and IL-10) in the treatment group were significantly reduced compared to the control at all the timepoints (*p* < 0.01, Figure 6A–F).

The ELISA results showed that the levels of pro-inflammatory cytokines in the duodenum of the treatment groups were significantly higher than the control groups at all the timepoints. However, there was no significant difference between the treatment and control groups for IL-1β at D60 and TNF-α at D28 (*p* > 0.01, *p* > 0.05, Figure 6G–L).

#### 2.4.2. mRNA and Protein (ELISA) Expression Levels of Pro- and Anti-Inflammation-Related Cytokines in the Jejunum of *A. adenophora*-Fed Rats

**Figure 7:**
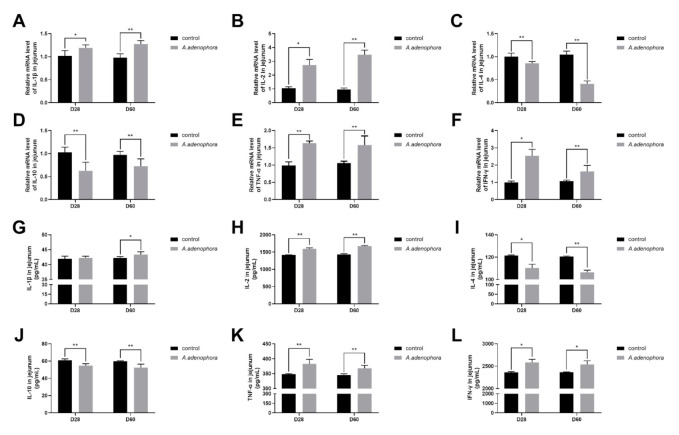
mRNA and protein (ELISA) expressions of pro- and anti-inflammation-related cytokines in the jejunum of rats from the control and *A. adenophora* groups after 60 days of experimental feeding. (**A**–**F**) mRNA expression of pro- and anti-inflammatory cytokines. (**G**–**L**) Protein (ELISA) expression of pro- and anti-inflammatory cytokines. Bars represent means ± SD for eight rats per treatment. Within the same day (D), bars with ** or * differ significantly (*p* < 0.01 or *p* < 0.05). Control = rats were fed only basal diet. *A. adenophora* = rats were fed a mixture of basal diet and leaf powder of *A. adenophora* in a 7:3 ratio.

In the jejunum, the gene expression level of all the related pro-inflammatory cytokines (IL-1β, IL-2, TNF-α, and IFN-ϒ) of the treatment groups were significantly higher as compared to the control group at all timepoints (*p* < 0.01 or 0.05). In addition, the gene expression levels of the anti-inflammatory cytokines (IL-4 and IL-10) in the treatment groups were reduced significantly as compared to the control group at all the timepoints (*p* < 0.05) (Figure 7A–F).

The jejunum ELISA results obtained in the jejunum also showed an increase in the pro-inflammation cytokines and a reduction in the anti-inflammation cytokine levels in the treatments groups compared to the control at all the timepoints; however, at D28, we found no significant difference in the IL-1β levels between the treatment groups and the control group (*p* < 0.01 or 0.05, Figure 7G–L).

#### 2.4.3. mRNA and Protein (ELISA) Expressions Levels of Pro- and Anti-Inflammation-Related Cytokines in the Ileum of *A. adenophora*-Fed Rats

**Figure 8:**
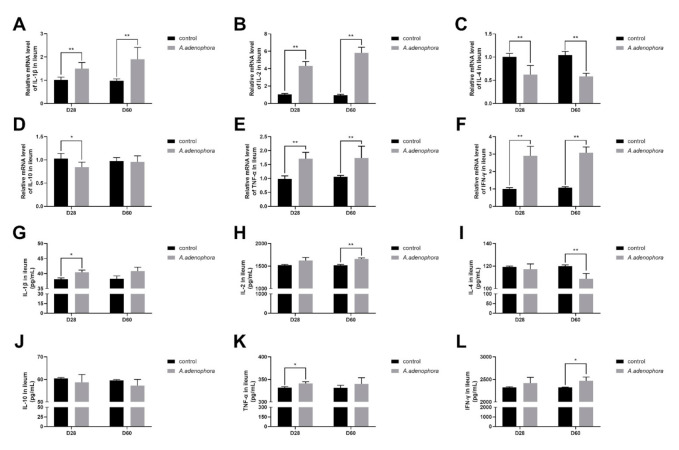
mRNA and protein (ELISA) expressions of pro- and anti-inflammation-related cytokines in ileum of rats from the control and *A. adenophora* groups after 60 days of experimental feeding. (**A**–**F**) mRNA expression of pro- and anti-inflammatory cytokines. (**G**–**L**) Protein (ELISA) expression of pro- and anti-inflammatory cytokines. Bars represent means ± SD for eight rats per treatment. Within the same day (D), bars with ** or * differ significantly (*p* < 0.01 or *p* < 0.05). Control = rats were fed only basal diet. *A. adenophora* = rats were fed a mixture of basal diet and leaf powder of *A. adenophora* in a 7:3 ratio.

In the ileum, the levels of pro-inflammatory cytokines of the treatment groups were significantly higher than the control group at all the timepoints (*p* < 0.01). In addition, the levels of the anti-inflammatory cytokines were reduced in the treatment groups as compared to the control; however, there was no significant difference between the treatment and control groups for IL-10 at D60 (*p* < 0.01 or *p* < 0.05, Figure 8A–F).

Furthermore, the ELISA results showed that the levels of the pro-inflammatory cytokines were elevated, but no difference existed between the treatment and the control groups for IL-1β (at D60), IL-2 (at D28), TNF-α (at D60), and IFN-ϒ (at D28) (*p* > 0.01 or *p* > 0.05). The levels of IL-4 and IL-10 were decreased in the treatment groups as compared to the control groups at all the timepoints; however, only the level of IL-4 was significantly reduced in the treatment group compared to the control group at D60 (*p* < 0.01, Figure 8G–L).

#### 2.4.4. mRNA and Protein (ELISA) Expressions of Pro- and Anti-Inflammation-Related Cytokines in the Cecum of *A. adenophora*-Fed Rats

**Figure 9:**
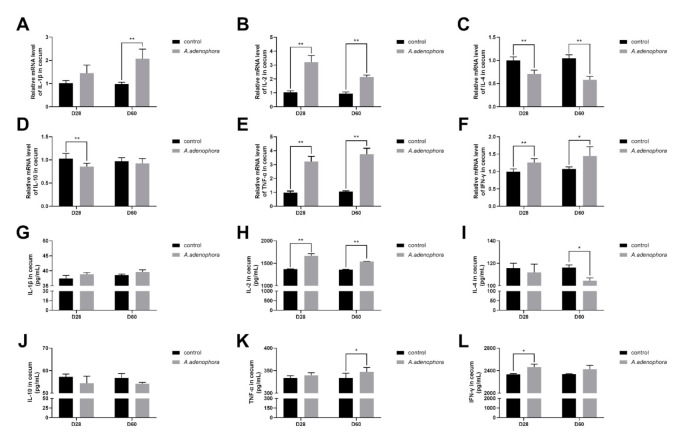
mRNA and protein (ELISA) expressions of pro- and anti-inflammation-related cytokines in cecum of rats from the control and *A. adenophora* groups after 60 days of experimental feeding. (**A**–**F**) mRNA expression of pro- and anti-inflammatory cytokines (**G**–**L**) Protein (ELISA) expressions of pro- and anti-inflammatory cytokines. Bars represent means ± SD for eight rats per treatment. Within the same day (D), bars with ** or * differ significantly (*p* < 0.01 or *p* < 0.05). Control = rats were fed only basal diet. *A. adenophora* = rats were fed a mixture of basal diet and leaf powder of *A. adenophora* in a 7:3 ratio.

The gene expression results in the cecal section of the large intestine showed that the levels of pro-inflammation cytokines in the treatment groups were higher than in the control groups; however, there was no significant difference recorded for IL-1β at D60. The levels of IL-4 and IL-10 cytokines were reduced in the treatment groups as compared to the control group; however, only IL-4 showed significant difference at D60 (*p* < 0.05, Figure 9A–F).

Furthermore, the ELISA results also showed similar trends as the mRNA expression results; however, there were no significant differences for pro-inflammatory cytokines IL-1β (at D8 and D60), TNF-α (at D28), and IFN-ϒ (at D60), and anti-inflammatory cytokines IL-4 (at D28) and 1L-10 (at D28 and D60), respectively (*p* > 0.01 or *p* > 0.05, Figure 9G–L).

#### 2.4.5. mRNA and Protein (ELISA) Expression Levels of Pro- and Anti-Inflammation-Related Cytokines in the Colon of *A. adenophora*-Fed Rats

**Figure 10:**
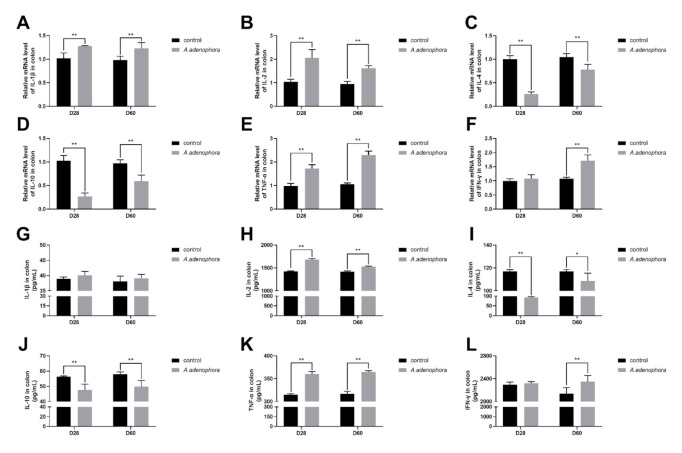
mRNA and protein (ELISA) expressions of pro and anti-inflammation-related cytokines in the colon of rats from the control and *A. adenophora* groups after 60 days of experimental feeding. (**A**–**F**) mRNA expression of pro- and anti-inflammatory cytokines. (**G**–**L**) Protein (ELISA) expression of pro- and anti-inflammatory cytokines. Bars represent means ± SD for eight rats per treatment. Within the same day (D), bars with ** or * differ significantly (*p* < 0.01 or *p* < 0.05). Control = rats were fed only basal diet. *A. adenophora* = rats were fed a mixture of basal diet and leaf powder of *A. adenophora* in a 7:3 ratio.

Colonic mRNA expressions of pro-inflammation cytokines were significantly increase in the treatment groups compared to the control group at all the timepoints (*p* < 0.01), except for cytokines TNF-α, which showed no difference at D28 (*p* > 0.01 or *p* > 0.05, Figure 10A–F). IL-4 and IL-10 cytokines were significantly reduced in the treatment group as compared to the control group at all timepoints (*p* < 0.01).

The protein levels of pro- (IL-1β, IL-2, TNF-α, and IFN-ϒ) and anti- (IL-4 and IL-10) inflammation cytokines showed similar trends as their mRNA expressions; however, there were no differences between the expressions of IL-1β (at D20 and D60) and IFN-ϒ (at D28) (*p* > 0.01 or *p* > 0.05, Figure 10G–L).

#### 2.4.6. mRNA and Protein (ELISA) Expressions Levels of Pro- and Anti-Inflammation-Related Cytokines in the Rectum of *A. adenophora*-Fed Rats

**Figure 11:**
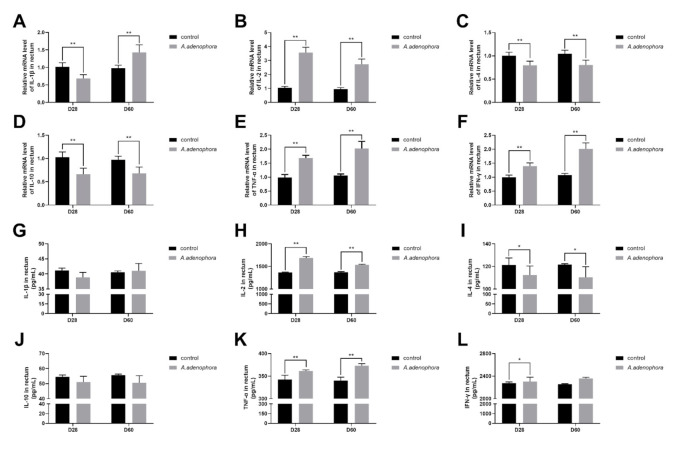
mRNA and protein (ELISA) expressions of pro- and anti-inflammation-related cytokines in the rectum of rats from the control and A. adenophora groups after 60 days of experimental feeding. (**A**–**F**) mRNA expression of pro- and anti-inflammatory cytokines (**G**–**L**) Protein (ELISA) expression of pro- and anti-inflammatory cytokines. Bars represent means ± SD for eight rats per treatment. Within the same day (D), bars with ** or * differ significantly (*p* < 0.01 or *p* < 0.05). Control = rats were fed only basal diet. *A. adenophora* = rats were fed a mixture of basal diet and leaf powder of *A. adenophora* in a 7:3 ratio.

The mRNA expression in the rectal tissues of rats fed *A. adenophora* showed that the pro-inflammation cytokines (IL-1β, IL-2, TNF-α, and IFN-ϒ) were elevated, whereas the anti-inflammatory cytokines (IL-4 and IL-10) decreased in the treatment groups compared with the control group at all timepoints (*p* < 0.01, Figure 11A–F). Similar trends were observed with the protein expression results; however, no difference occurred with cytokines IL-1β (at D28 and D60), IL-10 (at D28 and D60), and IFN-ϒ (at D60) (*p* > 0.01 or *p* > 0.05, Figure 11G–L).

## 3. Discussion

*A. adenophora* has toxic effects on intestinal tissues. In the present study, the pathological results obtained in this study showed that the ingestion of *A. adenophora* caused severe pathological changes on various sections of the intestine as compared to the control group. Complications like lymphocytosis, lymphocytic hyperplasia, lymphocytic proliferation, edema, necrosis, and inflammation were observed at various sections of the intestine of rats fed *A. adenophora,* except the control group. This was consistent with previous reports that observed pathological changes on organs such as the intestine, liver, and spleen after *A. adenophora* or its metabolites ingestion [6,21,22].

Villus height (V), crypt depth (C), and V:C ratio are three key morphological indicators of the overall health and functions of small intestine [23]. Villi are important structures in the small intestine that are responsible for nutrient absorption [24]. High villi height directly affects their capability of nutrient absorption in the intestine, as it increases surface area and hence promotes absorption [25]. In this study, it was observed that most sections of the small intestine in the treated rat groups had significantly shorter villi heights as compared to the control, indicating that *A. adenophora* could cause changes in the villi structure, thereby reducing its height.

Intestinal crypts are invaginations of the epithelium around the villi and are lined by epithelial cells that secrete enzymes. The base of the crypts is constantly dividing to maintain the structure of the villi; therefore, an increase in crypt depth would produce more developed villi [25]. The results obtained from this study indicated that the crypt depths of the treatment groups were all elevated. A study by Xue et al. [26] reported that deeper crypts indicate faster tissue turnover for villus renewal, which may be needed in response to inflammation caused by pathogens or their toxins. In addition, another study reported that reducing crypt depths of intestinal villi may lead to a reduction in the absorption of nutrients [27]. Therefore, on this account, it could be concluded that *A. adenophora* induces pathological damage or injury in the gut as confirmed by the histological scores in Figure 1. In addition, a greater V:C ratio suggests an increased nutrient absorption [23]. In this study, we also observed a decrease in the V:C ratio of the treatment groups compared to the control group, which confirmed the inhibiting ability of *A. adenophora* on gut function. The results from the histopathological and morphological analysis were similar to the reports on *Robinia pseudoacacia*, another toxin and invasive plant that is known to first cause pathological changes in the intestinal cells and structure as well as cause enteritis (inflammation of the small intestines) once it gets ingested by animals especially horses [28].

The intestinal intraepithelial lymphocytes (IELs) and Lamina propria leukocytes (LPLs) form the first line of the host immune defense system and play an essential role against infections caused by certain microorganisms and parasites such as *E. coli*, *Salmonella*, tape worm, and hook worm [29,30]. Various studies have reported that an increase in the levels of IELs indicates inflammation or other diseased conditions [31]. Therefore, the increase in IELs and LPLs counts in the treatment groups as compared to the control group could indicate that *A. adenophora* induced inflammation in the intestine of the rat. A similar result was reported by Sun et al. [6] that *A. adenophora* induces inflammation in the liver of mice.

sIgA is a predominant immunoglobulin in the mucosal system and is critical for protecting mucosal surfaces against toxins, viruses, and enteropathogens [32]. High sIgA levels may usually indicate that a patient’s body is overreacting in respect to immune response [33]. The core function of the gut immune system is the secretion of sIgA. The intrinsic layer of intestinal mucosa distributes about 80% of sIgA cells in the body, which are secreted by lymphocytes and plasma cells in the form of dimer or polymer [34]. The synthesis of sIgA is also related to the production of antigens and the distribution of cytokines in the intestinal environment [35]. The main function of sIgA in the intestine is to prevent the adhesion of pathogens to the intestinal mucosa surface, block the interaction between adhesion hormone and the surface of epithelial cells, neutralize enterotoxin in the gut, inhibit the movement of the pathogen in the intestinal mucosa epithelium, and also to maintain the integrity of the intestinal mucosa epithelium. The decrease of sIgA would reduce the anti-infective immune barrier function of intestinal mucosa to some extent and increase the interactions that co-exist between intestinal bacteria, endotoxin, and mucosal epithelial cells, as well as lead to bacterial translocation and endotoxin absorption [35]. Our results showed that the levels of intestinal sIgA were elevated in the treatment groups indicating that *A. adenophora* caused gut infection or inflammation that triggered overexpression of immune response. This result was consistent with findings of [33], who reported that toxins and pathogens could increase the sIgA levels in the body once they are ingested.

The intestinal barrier is a complex multilayer system, consisting of an external anatomical barrier and an inner functional immunological barrier [36]. The interplay of these two barriers maintains normal intestinal function and a stable intestinal environment [37]. Chemical markers (such as DAO and D-lactic acid) are important indicators of intestinal permeability. When the intestinal barrier function is damaged, these chemical markers get released into the blood. DAO is a kind of intracellular enzyme that catalyzes the oxidation of deamine, putrescine, and histamine [38]. More than 95% of DAO exists in mammalian and human small intestinal mucosa and ciliated epithelial cells, with the highest activity occurring in the jejunum and ileum [39]. Therefore, in this study, the high level of DAO obtained in the serum reflects the level of mechanical injury of the small intestine. D-LA is the product of anaerobic metabolism of glucose in the body. The level of D-LA is stable and low in the physiological state of the body. However, in the case of ischemia, the intestinal aerobic metabolism decreases and the anaerobic metabolism increases, and its content rises sharply, which eventually leads to lactic acid poisoning. In addition, mammals lack that special enzymatic system to decompose D-LA. Therefore, this index is often used in clinical and actual production to reflect the anoxia of intestinal body, intestinal mucosal damage, and intestinal mechanical barrier [40]. Once the intestinal barrier is disrupted, the intestinal epithelium releases DAO and D-LA into the bloodstream, and these are employed as indicators for intestinal integrity [41,42]. Our results showed that the levels of both DAO and D-LA were elevated in the *A. adenophora*-treated groups as compared to the control group, and this indicated that *A. adenophora* could cause destruction of intestinal integrity and epithelial function. This results were consistent with the study of Yang et al. [43], who reported that lipopolysaccharide (LPS) an endotoxin could elevate the levels of DAO and D-LA in the serum of broilers.

The intestinal barrier function relies on special components such as mucosal structural components (e.g., a hydrated gel composed of mucins) and intercellular junctions (e.g., tight junction (TJ) and adherens junction) [44,45]. The TJs are crucial for determining paracellular permeability [45,46]. Occludins are tight junction proteins that form integral membrane proteins crucial for tight junctions, and its adherens are involved in cell-to-cell adhesion as well as communication [47]. Occludin is composed of multiple gene families and plays different roles according to the cell type in different situations [48]. Some studies have shown that occludin plays a key role in regulating the passage of macromolecule through the gut as well as regulating the permeability of intestinal mucosa [49]. Occludin protein plays an indispensable role in the formation of intestinal mucosal barrier and the expression of occludin is significantly reduced in some intestinal diseases such as Crohn’s disease and ulcerative colitis [50], suggesting that occludin could protect the intestinal barrier. Leaky gut is associated with reduced expression of tight junction proteins such as occludin [51]. We observed a reduction in occludin levels in the treatment groups as compared to the control, and this indicates that *A. adenophora* could cause leakage in the gut, thereby reducing its immune integrity. This result was consistent with the findings of Romero et al. [52], who reported that mycotoxins such as aflatoxin B1 (AFB1), fumonisin B1 (FB1), ochratoxin A (OTA), and T-2 toxin (T2) could reduce the mRNA and protein levels of the tight junction constituents such as claudin-3, claudin-4, and occluding in human Caco-2 cells.

Studies have shown that in situations of body poisoning, there is a protective reaction against infection or injury, namely inflammatory reaction. Cytokines are endogenous mediators of the immune system that play major roles in controlling the occurrences of inflammatory reaction [53]. For instance, in cases of acute toxicity (such as toxin metabolism), the levels of IL-6, IL-2, tumor necrosis factor, and other inflammatory factors increase dramatically [54]. The intestinal barrier of mammals performs both protective (the deeper layers of the intestinal wall) and regulatory (tightly regulates the passage of pro-inflammatory molecules, microorganisms, toxins, and antigens) roles to promote their intestinal structure and integrity [55]. Cytokines performs an important biological function in immunomodulatory function, antiviral response, and inflammatory reaction via being organized within a stratified regulatory network through complex interactions, which may reflect anti-inflammation ability and antiviral response [56]. Acute inflammation causes tissue damage [57,58] due to factors such as high expression of pro-inflammation cytokines (IL-1β and TNF-α) and permitting damages to intestinal epithelial cells via autocrine or paracrine action [59,60]. Our results showed that both the mRNA and protein (ELISA) expressions levels of pro-inflammation cytokines (IL-1β, IL-2, TNF-α, and IFN- ϒ) were increased in rats fed A. adenophora leaf powder. We therefore speculated that *A. adenophora* could cause inflammation to the gut by promoting the release of pro-inflammation cytokines that directly affect the integrity of the intestinal immune barrier. This result was consistent with the work of [59], who reported that *A. adenophora* could significantly increase the mRNA expression levels of IL-2, IL-6, TNF-α, IFN-β, and IFN-γ mRNA in the epithelial cells and macrophages of Saanen dairy goats. We also observed a decrease in the production of anti-inflammatory cytokines (IL-4 and IL-10) in the intestinal tissue in our study. Therefore, we concluded that *A. adenophora* could cause inflammation in the gut by promoting the release of pro-inflammatory-related cytokines such as TNF-α, IFN-β, and IFN-γ.

To sum up, even though our group did not perform chemical analyses of the *A. adenophora* samples, the analyses performed in other studies have previously revealed an abundance of three important sesquiterpenes, i.e., 2-deoxo-2-(acetyloxy)-9-oxoageraphorone (DAOA), 9-oxo-agerophorone (OA), and 9-oxo-10,11-dehydro-agerophorone (ODA, Euptox A) in the plant leaves [22,61,62]; therefore, based on the reported literature, it would seem plausible these sesquiterpenes could be the main causative agents for the intestinal toxicity we have observed in our study; however, this needs further studies to ascertain the specific toxic metabolites that cause the gut toxicity.

## 4. Conclusions

*A. adenophora* ingestion leads to the secretion of pro-inflammatory cytokines, intracellular contents, and induces extensive inflammatory responses [7,63]. Our study showed that *A. adenophora* could promote intestinal inflammation, disrupt intestinal structure and integrity, and also reduce the efficiency of the intestinal immune barrier. This study will serve as a scientific tool for further studies on the toxic effects of *A. adenophora* on the intestine, especially on the effects caused by its specific secondary metabolites on intestinal microbiota and their metabolites, as well as their underlying mechanism in inducing intestinal toxicity.

## 5. Materials and Methods

### 5.1. Plant Material

*Ageratina adenophora* were collected from Dechang in Sichuan Province (102°15′20″ E and 27°20′11″ N, Elevation 2152 m). The samples were confirmed as *A. adenophora* by Professor Chao Hu of the department of Botany, Sichuan Agricultural University, Chengdu, China. For the experimental feed preparation, the leaf samples were air-dried and then ground into a fine powder using a CW700 grinding machine (Pharmaceutical Machinery Co., Shan Dong, China). Sizing analyses showed that the mean diameter of the powder particles was 500 μm. Thereafter, the A. adenophora powder was mixed uniformly with normal feed powder (Chengdu Dashuo, Table 2) in a ratio of 3:7. Both basal and experimental diets were shaped into a pellet with a cylinder shape tube with diameter of 2 cm and height of 5 cm as described by Sun et al. [6].

### 5.2. Animals Experiment and Plant Materials

In total, 48 male Sprague-Dawley (SD) rats (7 weeks, ≈200 g) were obtained from the Dashuo Experiment Animal Co. (Chengdu, Sichuan Province, China). All rats were housed in specific pathogen-free facilities maintained at 22–24 °C with a 40–60% relative humidity and a 12-hour light:dark cycle. After 7 days of adaptation, the rats were randomly allocated into two groups, i.e., a control (*n* = 24) that was fed a basal diet at 10 g/100 g BW, and an *A. adenophora* treatment group (*n* = 24) that also received 10 g/100 g BW of the experimental diet. The experimental period was 60 days. The dose of the *A. adenophora* powder used in this experiment was based on a pilot experiment that indicated that feeding mice with a mixture of 30% *A. adenophora* leaf powder and 70% basal diet for a period of 60 days did not affect feed intake [64,65]. Throughout the experiment, drinking water was provided to rats ad libitum.

Rats were observed over the course of the entire 60-day period; body weights were taken weekly, whereas behavior, illness, and mortality parameters were observed and recorded throughout the entire period. On day 28 and 60 respectively, eight (8) rats were randomly selected from each group, fastened (12 h), weighed, and euthanized by CO_2_ asphyxiation. Prior to the killing, blood samples were collected from the abdominal aorta and were transferred into anticoagulant-coated tubes; thereafter, the blood samples were centrifuged to obtained serum that was stored at −80 °C until further analysis. Intestinal tissue was then aseptically removed and 1 cm in length of the duodenum tissue (distanced 2 cm from the pylorus), jejunum tissue (in the middle segment of jejunum), ileum tissue (collected from the terminal ileum), cecum tissue (0.5 cm to blind end), colon tissue (8 cm proximal to the anus), and rectal tissue (1 cm proximal to the anus) were collected. Furthermore, histopathological assay, cytokines analyses, and nucleic acid extraction were performed.

### 5.3. Ethics Statement

This study was approved by the Animal Care and Use Committee of Sichuan Agricultural University (Approval No.: 2012-024). All animal operation and procedures were conducted according to the approved guidelines and were in accordance with the International Guide for the Care and Use of Laboratory Animals.

### 5.4. Histopathological Evaluation

Parts of the intestinal tissues were temporarily stored in a 10% buffered neutral formalin solution for 12 h and then embedded in paraffin. Thereafter, HE staining and AB-PAS staining were performed according to the manufacturer’s instructions (Servicebio, Wuhan, China). For HE, we examined the pathological morphology and gave histologic injury scores to all the intestinal tissues using the criteria outlined in Table 3; five horizons were selected randomly for intraepithelial leukocytes (IELs) and lamina propria leukocytes (LPLs) counting, completed villus-crypt structures were randomly selected, and villus height, width, and crypt depth were measured using Image Pro Plus 6.0 (Media Cybernetics, Bethesda, MD, USA), the same software that was used to inspect the distribution and number of goblet cells by AB-PAS staining. A total of 8 rats/group were evaluated; for each rat, 3 stained slices were examined.

### 5.5. Enzyme-Linked Immunosorbent Assay

Parts of the intestinal tissues were washed with PBS, 0.1 g of the sample tissue was weighed and homogenized with 0.9 mL ice-cold PBS in a glass homogenizer, and then the mixture was centrifuged (3000 rpm, 20 min) to obtain the supernatant. To evaluate the extent of immune injury in the intestine, the supernatants were used to determine the concentrations of secretory IgA (sIgA), IL-1β, IL-2, IL-4,TNF-α, and IFN-ϒ, and the evaluations were performed using a commercial rat ELISA kit (Jingmei Biological Technology, Jiangsu, China), respectively, and then the concentrations of diamine oxidase (DAO) and D-Lactic acid (D-LA) were determined in the blood serum using commercial ELISA kits (Jingmei Biological Technology, Jiangsu, China) to assess the degrees of gut mucosal barrier damage according to the manufacturer’s instructions. sIgA was measured as μg/ml and cytokines pg/mL. The level of sensitivity of each kit was 0.1μg/mL for sIgA and 0.1 pg/mL for each cytokine.

### 5.6. Immunohistochemistry Assay

The intestines were carefully dissected from the rats, washed with cold PBS (pH 7.2–7.4), and then fixed overnight in 4% paraformaldehyde and embedded in paraffin wax after dehydration. The paraffin-embedded intestinal tissues were sliced into 4 µm sections and then dewaxed in xylene, followed by rehydrating through a graded series of ethanol solutions. Endogenous peroxidase was blocked by incubating with 3% H_2_O_2_ in methanol for 10 min. After, heat-induced antigen retrieval was performed using citrate buffer (10 mM, pH = 6.0). The sections were blocked with goat serum (Sigma, St. Louis, MO, USA) for 15 min, and incubated overnight at 4 °C with a rabbit monoclonal anti-rat occludin antibody (ab216327, Abcom, dilution 1:200). Each section was rewarmed at 37 °C for 1 h and washed in PBS for 10 min, followed by incubation with biotin-tagged anti-rabbit second antibody (SP9001, ZSGB-BIO, Beijing, China) at 25 °C for 15 min. Furthermore, horseradish peroxidase-labeled streptavidin working solution (S-A/HRP) was added and after 15 min, the mixture was slowly flushed with PBS. Finally, freshly prepared DAB chromogenic solution was added, then after 6 min, hematoxylin was also added for 20 s. The slides were examined for positive staining and were subjected to optical density analysis with a Leica DM-1000 microscope (Leica Microsystems Imaging Solutions Ltd., Cambridge, UK) using Image Pro Plus 6.0 (Media Cybernetics, Bethesda, MD, USA), and three visual fields were randomly selected in each section.

### 5.7. RNA Extraction and Quantitative Real-Time PCR Analysis

The intestinal tissues stored in the −80 °C were subjected to RNA extraction. Every intestinal segment was opened longitudinally, cleaned by PBS, snap frozen in liquid nitrogen, and grinded into powder with a mortar and pestle. The total RNA from each sample was extracted with an Animal Total RNA Isolation Kit (Sagon Biotech, Shanghai, China) following the manufacturer’s instructions. RNA concentrations were determined by absorbance at 260 nm in a UV spectrophotometer (Thermo Fisher Scientific, Waltham, MA; OD260/280 ≈ 1.9–2.0). After 2 μg RNA was reverse transcribed into 20μL cDNA using a PrimeScrip RT reagent kit (Takara, Tokyo Japan), quantitative real-time PCR was performed by using a CFX96 PCR detection system (BioRad, Hercules, CA, USA) with a SYBR Premix ExTaq (Takara). The conditions of PCR were as follows: 95 °C for 5 min, then 40 cycles of 95 °C, 15 s for denaturation, 60 °C, 60 s for annealing at 70 °C, 25 s for extension. Each qRT-PCR reaction was performed with volumes of 15 µL containing 6.25 µL TB Green TM Premix (Takara), 0.3 µL forward and reverse primers, 1.5 µL cDNA, and 6.65 µL DNase/RNase-Free Deionized Water (Tiangen, Beijing, China). The gene primers used in this experiment are shown in Table 4. The relative expression levels were normalized by internal reference Beta-actin (β-actin) through the 2^−ΔΔCt^ method.

### 5.8. Statistical Analysis

The Shapiro–Wilk test was used to test the normality of the data. All the variables that met the normality criteria, a one-way analysis of variance (ANOVA) was used to detect differences among the groups (Tukey’s test). Data sets that did not display normal distribution were analyzed by non-parametric tests (Kruskal–Wallis test). The data were represented as means ± standards deviations and analyzed with the Statistical Program for Social Sciences software for Windows (SPSS, version 17.0; SPSS Inc., Chicago, IL, USA). In all cases, * *p* < 0.05, or ** *p* < 0.01 were considered significant.

## Figures and Tables

**Table 1:** Effects of *A. adenophora* on villi height, crypt depth, and the villi height ratio/crypt depth (*v*/*c*) of the small intestinal sections in rats (μm; *n* = 8).

Intestinal Segment	Index	Treatments	D28	D60
duodenum	Villus height	Control	488.63 ± 35.29 ^A^	485.42 ± 25.87 ^A^
		*A. adenophora*	390.11 ± 28.23 ^B^	384.76 ± 26.39 ^B^
	crypt depth	Control	78.88 ± 15.50 ^B^	74.91 ± 14.37 ^B^
		*A. adenophora*	107.51 ± 6.72 ^A^	135.81 ± 35.53 ^A^
	Villus height/crypt depth ratio	Control	6.32 ± 0.54 ^A^	6.41 ± 0.49 ^A^
		*A. adenophora*	3.51 ± 0.40 ^B^	3.14 ± 0.87 ^B^
jejunum	Villus height	Control	400.55 ± 10.96 ^A^	418.26 ± 12.75 ^A^
		*A. adenophora*	275.63 ± 40.78 ^B^	258.43 ± 28.20 ^B^
	crypt depth	Control	89.72 ± 11.73 ^B^	89.34 ± 15.15 ^B^
		*A. adenophora*	100.18 ± 10.32 ^A^	193.43 ± 25.19 ^A^
	Villus height/crypt depth ratio	Control	4.49 ± 0.57 ^A^	4.69 ± 0.48 ^A^
		*A. adenophora*	2.82 ± 0.43 ^B^	1.36 ± 0.23 ^B^
ileum	Villus height	Control	367.09 ± 25.05 ^A^	369.49 ± 27.86 ^A^
		*A. adenophora*	205.11 ± 36.04 ^B^	267.88 ± 24.93 ^B^
	crypt depth	Control	71.32 ± 11.05 ^B^	80.82 ± 12.32 ^B^
		*A. adenophora*	107.79 ± 6.80 ^A^	163.53 ± 13.79 ^A^
	Villus height/crypt depth ratio	Control	5.03 ± 0.39 ^A^	4.97 ± 0.75 ^A^
		*A. adenophora*	2.33 ± 0.34 ^B^	1.66 ± 0.24 ^C^

Table 1 is represented as means value ± standard deviation (SD). Columns with different superscripts are statistically significant (*p* < 0.05 or *p* < 0.01). Control = rats were fed only basal diet. *A. adenophora* = rats were fed a mixture of basal diet and leaf powder of *A. adenophora* in a 7:3 ratio.

**Table 2:** Composition of the basal diet.

Ingredients	Content %
Water	10
Crude protein	18
Crude fat	4
Crude fiber	5
Crude ash	8
Calcium	1.5
Phosphorus	1
Lysine	0.82
Methionine + Cystine	0.53

**Table 3:** Criteria for histological scoring of damaged intestinal tissues.

Appearance	Score
Edema	0 or 1 (absent or present)
Angiogenesis	0 or 1 (absent or present)
Muscle thickening	0, 1, 2, or 3 (absent, mild→severe)
Cellular infiltration	0, 1, 2, or 3 (absent, mild→severe)
Loss of mucosal architecture	0, 1, 2, or 3 (absent, mild→severe)

**Table 4:** Primers used for the real-time PCR analysis.

Gene Name	Primer	Sequence (5′ and 3′)	Product Length (bp)	Annealing Temperature (°C)	Sequence Number
IL-1β	Forward	GCAGCTTTCGACAGTGAGGA	86	55.0	NM_031512.2
	Reverse	CCCAAGTCAAGGGCTTGGAA			
IL-2	Forward	TCTGCAGCGTGTGTTGGATT	143	52.38	NM_053836.1
	Reverse	CTGGCTCATCATCGAATTGGC			
IL-4	Forward	TGTAGAGGTGTCAGCGGTCT	70	60.25	NM_201270.1
	Reverse	TCAGTGTTGTGAGCGTGGAC			
IL-10	Forward	CTGGCTCAGCACTGCTATGT	86	60.11	NM_012854.2
	Reverse	GCAGTTATTGTCACCCCGGA			
TNF-α	Forward	CTCAAGCCCTGGTATGAGCC	90	60.18	NM_012675.3
	Reverse	CTTGGGCAGGTTGACCTCAG			
IFN-γ	Forward	CATCGCCAAGTTCGAGGTGA	87	60.00	NM_138880.3
	Reverse	TCTGGTGACAGCTGGTGAATC			
β-actin	Forward	ACGGTCAGGTCATCACTATCG	112	65.20	NM_031144.3
	Reverse	GGCATAGAGGTCTTTACGGATG			

## Data Availability

The data presented in this study are available on request from the corresponding authors.
